# Supplementary material for: The Chx10-Traf3 Knockout Mouse as a Viable Model to Study Neuronal Immune Regulation
Source: Cells. 2021 Aug 12;10(8):2068. doi: 10.3390/cells10082068 (PMC8391412; doi:10.3390/cells10082068)
Supplement: Supplementary file 1 [file cells-10-02068-s001.zip › cells-1305600-supplementary.pdf]

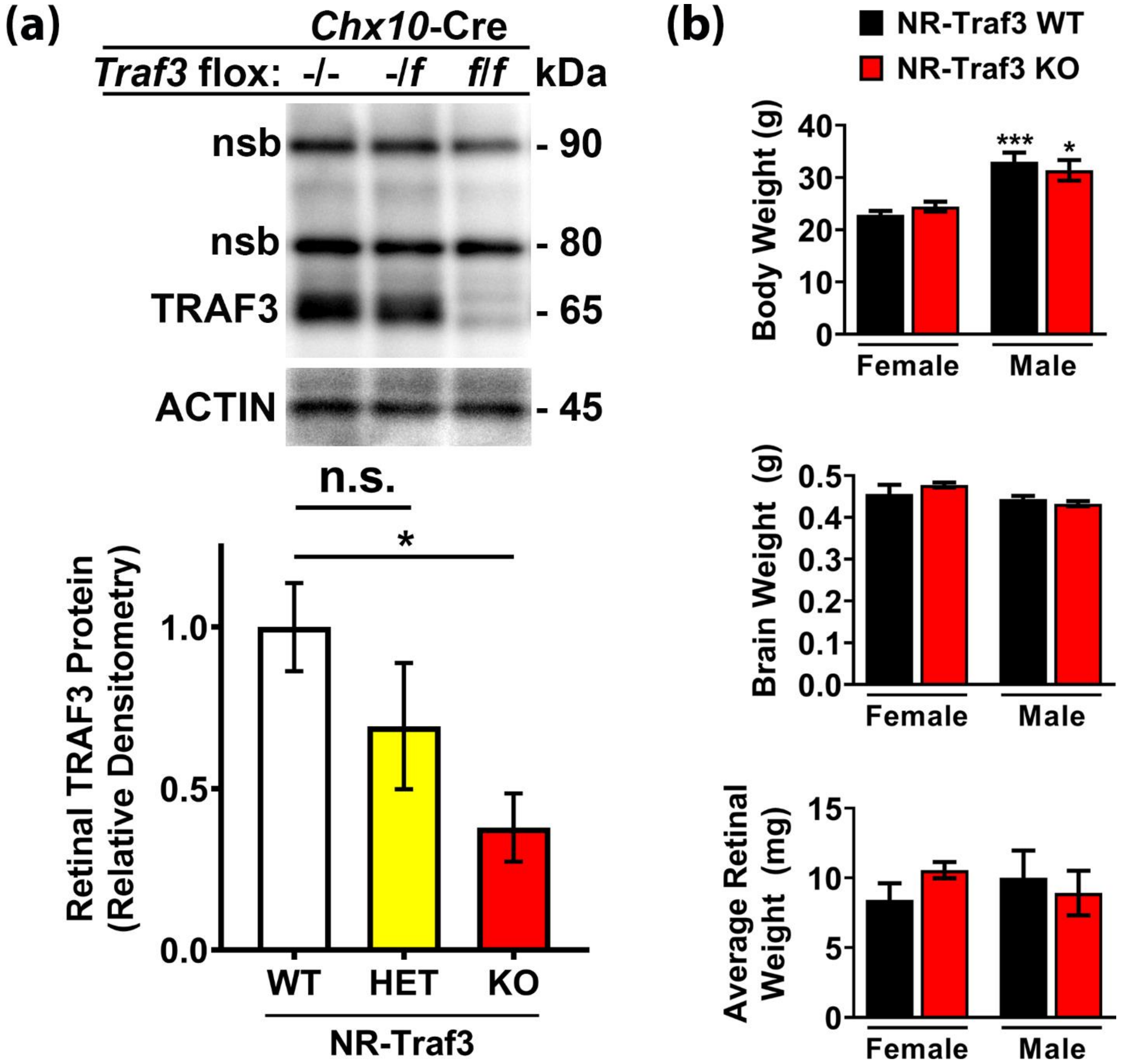

Figure S1. Effect of NR-*Traf3* depletion on retinal TRAF3 protein expression, body weight, brain tissue weight, and average retinal tissue weight. **(a)** Representative Western blot and corresponding quantitative relative densitometry data showing dose-dependent reduction of TRAF3 protein in *Chx10*-Traf3 wildtype (WT), heterozygous (HET), and knockout (KO) whole retinal tissue extracts. β-actin was used as loading control and for normalization of TRAF3 protein expression. *Traf3* flox -/-, -/f, and f/f indicate *Chx10*-Cre positive NR-*Traf3* WT, HET, and KO mice, respectively. Data are mean ± SEM and were analyzed via 1-way ANOVA with Dunnett's multiple comparisons post-hoc test (Genotype effect: * $p<0.05$; WT vs. HET, $p=0.1954$; WT vs. KO, $p=0.0179$; N=3). **(b)** Total body weight, total brain tissue weight, and average retinal tissue weight for adult female and male NR-*Traf3* WT and KO mice. Average retinal weight reflects individual mouse average retina weight calculated from both right and left eyes, which were then combined for group averaging. An expected body weight difference was observed between females and males, where total female body weight was lower than that of males (2-way ANOVA; Sex effect: *** $p<0.001$, * $p<0.05$; N=5). There were no other sex- or genotype-dependent differences in tissue weights. Data are mean ± SEM.

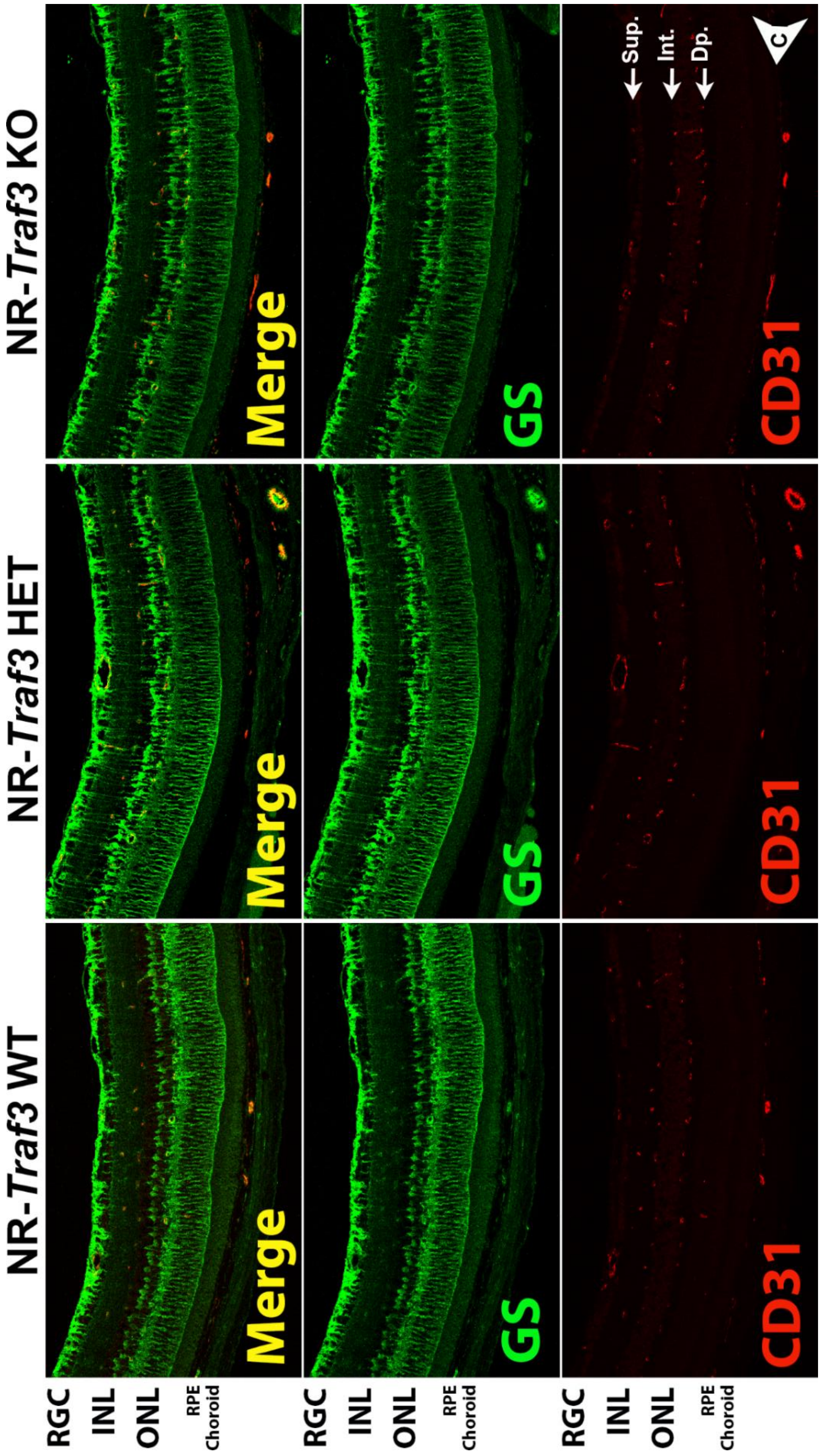

Figure S2. Normal Müller glial and vascular stratification occurs in mouse retinal tissue with NR-*Traf3* depletion. Immunofluorescent labeling of adult murine retinal Prefer-fixed retinal tissue sections stained with Müller cell-specific marker glutamine synthetase (GS) and endothelial cell-specific CD31 (N=4). Müller glia processes span the length of the retina from the internal limiting membrane of the RGC layer to the ONL. The larger choroidal vessels posterior to the RPE appeared normal (C, white arrowhead). All 3 layers of the inner retinal vasculature were also present and are visualized by CD31 staining in vessel cross sections in the superficial, intermediate, and deep vascular plexi (Sup., Int., and Dp. white arrows, respectively). RGC, retinal ganglion cell layer; INL, inner nuclear layer; ONL, outer nuclear layer; RPE, retinal pigment epithelial layer.

Table S1. Detailed SD-OCT analysis for NR-*Traf3* adult mouse retinal structure and lamination. This table corresponds to Figure 3 of the main text, and includes numerical data (micron (µm) units) for NR-*Traf3* WT and KO "Total Retinal Thicknesses (TRT)," as well as that for NR-*Traf3* HET animals. As described in Figure 3, TRT was determined for 8 Outer (purple) and Inner (orange) retinal superior (S), temporal (T), inner (I), and nasal (N) zones via calculation of the distance between boundaries 1 (inner limiting membrane) and 10 (Verhoeff's membrane), which were manually determined. Additionally, the table also includes data for the 3 major retinal depth sudivisions (IRT, MRT, and ORT), as well as data for the smaller retinal layers comprising each subdivision (IRT=RNFL+IPL+INL; MRT=OPL+ONL; ORT=IS+OS+RPE). Each layer thickness was determined similarly as described for TRT, except using their own corresponding boundary designations. Numerical values represent the OD/OS group averages, which were obtained from individual mouse OD/OS averages, and are reported as mean ± SEM (N=6; 1-way ANOVA, *p<0.05).

	**Retinal Zones**							
	Outer				***Inner***			
**TRT**	S	T	I	N	S	T	I	N
WT	220.7 ± 1.3	220.4 ± 1.8	223.8 ± 1.6	220.8 ± 1.5	207.2 ± 2.1	209.3 ± 2.3	217.8 ± 2.2	210.9 ± 2.7
HET	215.5 ± 2.5	216.2 ± 2.6	219.0 ± 2.6	214.5 ± 2.2	204.5 ± 2.4	202.7 ± 2.3	209.8 ± 1.6	202.2 ± 1.3*
KO	220.5 ± 2.5	220.3 ± 2.0	222.0 ± 1.7	219.0 ± 1.8	208.7 ± 3.0	205.8 ± 1.6	214.0 ± 2.6	208.3 ± 2.1
**IRT**								
WT	93.7 ± 0.9	93.8 ± 1.2	94.3 ± 1.1	93.3 ± 0.9	86.8 ± 1.6	87.7 ± 1.4	93.3 ± 1.4	89.4 ± 1.6
HET	90.7 ± 1.3	90.7 ± 1.4	92.2 ± 1.8	89.7 ± 1.2	85.0 ± 2.0	84.0 ± 1.2	89.5 ± 1.0	84.3 ± 1.0
KO	93.8 ± 1.2	93.5 ± 1.1	93.8 ± 0.9	92.5 ± 1.1	87.7 ± 2.4	86.5 ± 1.1	92.7 ± 1.4	88.8 ± 1.4
**RNFL**								
WT	11.0 ± 0.3	10.2 ± 0.5	10.2 ± 0.6	10.6 ± 0.6	10.1 ± 0.1	10.8 ± 0.2	10.8 ± 0.5	11.2 ± 0.2
HET	11.3 ± 0.2	11.2 ± 0.3	11.2 ± 0.2	10.7 ± 0.2	10.5 ± 0.4	11.5 ± 0.2	12.2 ± 0.2	11.0 ± 0.3
KO	11.0 ± 0.3	11.3 ± 0.3	11.2 ± 0.3	10.5 ± 0.5	10.5 ± 0.3	11.3 ± 0.2	11.3 ± 0.4	11.5 ± 0.3
**IPL**								
WT	59.8 ± 0.8	62.3 ± 2.4	60.9 ± 1.4	60.6 ± 1.1	63.5 ± 1.4	61.6 ± 1.6	62.6 ± 1.7	60.7 ± 1.0
HET	57.5 ± 1.3	59.0 ± 1.6	60.7 ± 2.6	58.8 ± 2.0	61.8 ± 1.9	57.2 ± 1.2	59.3 ± 1.1	58.2 ± 1.1
KO	60.7 ± 1.3	61.0 ± 1.4	60.8 ± 1.1	61.0 ± 1.4	63.7 ± 1.6	60.5 ± 1.3	63.0 ± 1.8	60.3 ± 1.2
**INL**								
WT	23.5 ± 0.6	21.9 ± 0.8	24.3 ± 0.8	22.9 ± 1.0	15.8 ± 1.2	18.2 ± 0.9	21.2 ± 1.4	19.5 ± 1.2
HET	22.3 ± 0.3	21.3 ± 0.7	21.2 ± 1.1	20.8 ± 0.8	15.2 ± 0.6	17.3 ± 0.3	19.0 ± 0.6	17.0 ± 0.3*
KO	23.2 ± 0.5	21.8 ± 0.9	22.8 ± 0.7	22.2 ± 0.6	16.5 ± 1.1	16.5 ± 0.6	19.5 ± 0.6	18.7 ± 0.3
**MRT**								
WT	70.2 ± 0.3	68.8 ± 1.0	71.0 ± 0.6	70.4 ± 0.6	66.0 ± 0.7	65.3 ± 0.5	66.8 ± 1.3	65.5 ± 0.6
HET	68.2 ± 0.9	67.7 ± 1.0	70.2 ± 0.9	68.2 ± 1.0	64.7 ± 1.1	63.3 ± 1.0	64.5 ± 0.8	63.2 ± 0.9
KO	69.7 ± 0.8	69.5 ± 0.7	71.2 ± 0.7	69.8 ± 0.9	66.3 ± 1.0	64.7 ± 0.8	65.3 ± 1.3	64.7 ± 0.8
**OPL**								

WT	9.2 ± 0.2	9.0 ± 0.0	9.0 ± 0.0	8.8 ± 0.2	8.3 ± 0.3	8.8 ± 0.2	9.2 ± 0.2	9.2 ± 0.2
HET	9.0 ± 0.0	9.2 ± 0.2	9.3 ± 0.2	8.8 ± 0.2	8.8 ± 0.2	8.7 ± 0.2	9.3 ± 0.2	9.0 ± 0.3
KO	9.2 ± 0.2	9.0 ± 0.0	9.0 ± 0.0	9.2 ± 0.2	9.3 ± 0.3	9.0 ± 0.0	9.0 ± 0.0	9.0 ± 0.0
**ONL**								
WT	61.2 ± 0.2	60.1 ± 1.0	62.3 ± 0.7	61.6 ± 0.6	58.0 ± 0.8	57.1 ± 0.5	57.8 ± 1.1	56.7 ± 0.5
HET	59.0 ± 0.9	58.7 ± 1.0	61.3 ± 0.8	59.3 ± 1.1	56.7 ± 1.3	55.2 ± 1.0	55.5 ± 0.7	54.7 ± 0.8
KO	60.8 ± 0.8	60.5 ± 0.7	61.8 ± 0.6	61.2 ± 0.9	58.0 ± 1.0	56.2 ± 0.5	56.0 ± 1.2	56.0 ± 0.6
**ORT**								
WT	57.6 ± 0.8	58.3 ± 0.9	59.0 ± 0.7	58.0 ± 0.9	54.9 ± 0.6	57.2 ± 0.9	58.0 ± 1.2	56.4 ± 0.9
HET	57.5 ± 0.6	58.0 ± 0.6	57.3 ± 0.6	57.5 ± 0.7	55.5 ± 0.7	55.8 ± 0.5	56.3 ± 0.6	55.7 ± 0.7
KO	57.2 ± 0.5	57.8 ± 0.4	57.7 ± 0.7	56.8 ± 0.7	55.2 ± 0.7	55.2 ± 0.6	56.7 ± 0.5	55.7 ± 0.6
**IS**								
WT	9.2 ± 0.2	9.1 ± 0.7	9.2 ± 0.4	9.5 ± 0.8	9.3 ± 0.4	9.8 ± 0.6	9.4 ± 0.6	9.8 ± 0.5
HET	8.8 ± 0.4	9.8 ± 0.6	9.8 ± 0.5	9.5 ± 0.4	9.3 ± 0.2	10.2 ± 0.5	9.3 ± 0.4	9.8 ± 0.3
KO	9.0 ± 0.3	9.2 ± 0.5	9.3 ± 0.2	9.3 ± 0.4	9.0 ± 0.4	9.8 ± 0.4	9.8 ± 0.3	9.7 ± 0.4
**OS**								
WT	25.5 ± 0.4	26.3 ± 0.7	27.1 ± 0.8	26.2 ± 0.7	23.7 ± 0.7	24.3 ± 1.1	25.8 ± 0.8	24.8 ± 0.8
HET	25.2 ± 0.5	25.3 ± 0.8	25.2 ± 0.5	25.0 ± 0.6	24.0 ± 0.5	23.5 ± 0.6	23.8 ± 0.5	23.3 ± 0.6
KO	25.8 ± 0.5	26.0 ± 0.7	27.2 ± 0.6	25.8 ± 0.4	24.7 ± 0.8	23.5 ± 0.8	25.3 ± 0.6	25.0 ± 0.7
**RPE**								
WT	23.3 ± 0.5	23.6 ± 0.4	23.2 ± 0.7	22.9 ± 0.6	22.5 ± 0.6	23.3 ± 0.6	23.6 ± 0.8	22.6 ± 0.7
HET	23.5 ± 0.8	23.7 ± 0.6	23.3 ± 0.7	23.7 ± 0.8	22.8 ± 0.5	23.0 ± 0.6	23.5 ± 0.6	22.7 ± 0.8
KO	22.5 ± 0.6	22.7 ± 0.8	21.5 ± 0.8	22.3 ± 0.8	22.0 ± 0.6	22.5 ± 1.0	22.2 ± 1.1	21.7 ± 0.8

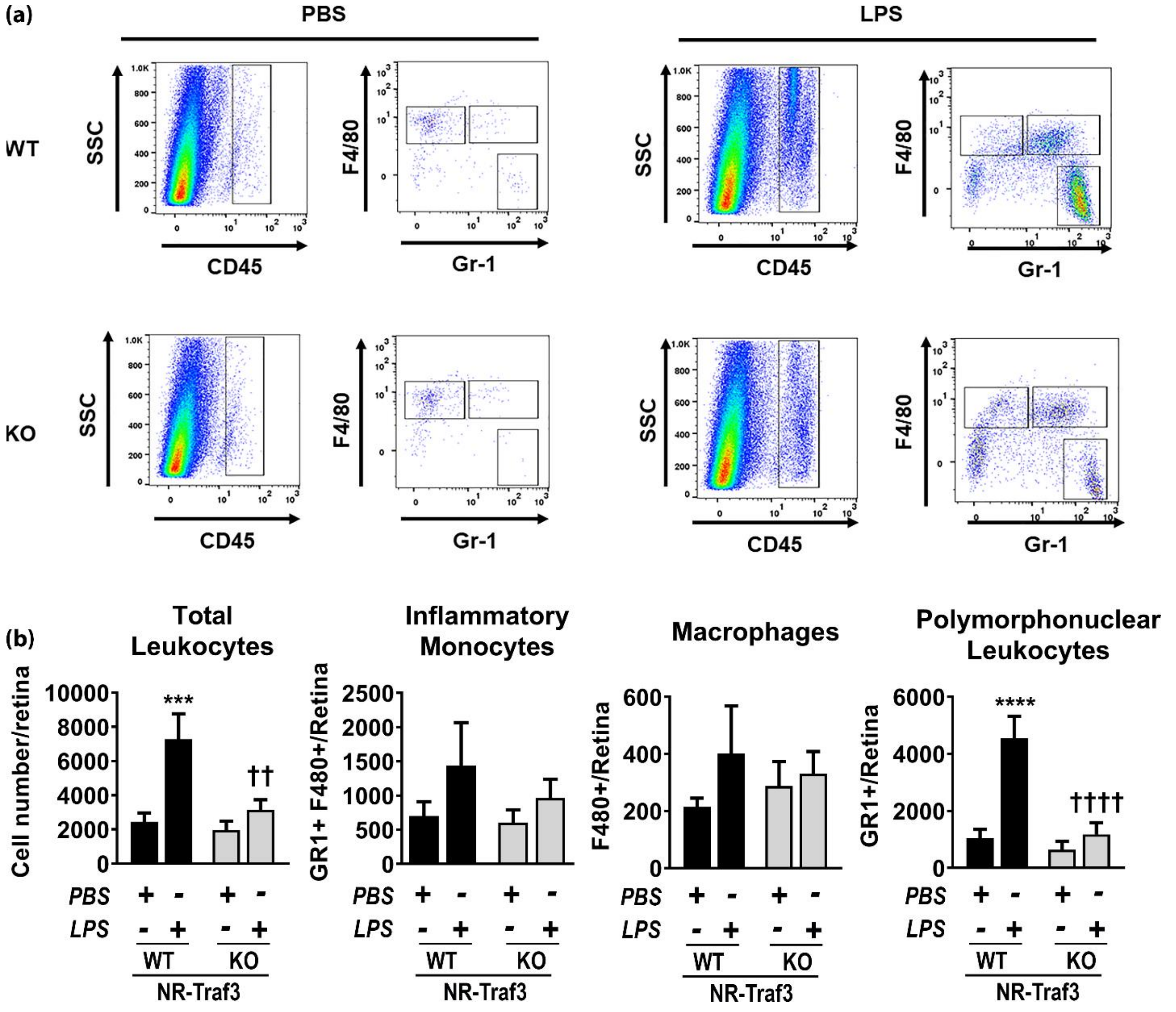

Figure S3. NR-*Traf3* promotes LPS-induced retinal immune cell infiltration (3-color). Three-color flow cytometry data showing **(a)** representative NR-*Traf3* WT and KO dot plots and **(b)** grouped histogram quantification of immune cells from whole retinal single cell suspensions following 24 hours intravitreal LPS administration. Data for **(b)** are mean ± SEM and were analyzed via 2-way ANOVA with Uncorrected Fisher's LSD multiple comparisons post-hoc test (LPS effect: **** $p<0.0001$, *** $p<0.001$; Genotype effect: ††††$p<0.0001$, ††$p<0.01$; N=3). Immune cell populations of interest are designated as: Total Leukocytes ($CD45^+$); Inflammatory Monocytes ($CD45^+GR1^+F4/80^+$); Macrophages ($CD45^+F4/80^+GR1^-$); Polymorphonuclear Leukocytes ($CD45^+GR1^+F4/80^-$).
